# Barriers and facilitators for healthcare providers to implement family-centered care in Parkinson's disease: a scoping review

**DOI:** 10.3389/fneur.2023.1231654

**Published:** 2023-08-25

**Authors:** Wen-Jing Sun, Ye-Jie Peng, Yan Liang

**Affiliations:** ^1^Department of Neurosurgery, West China Hospital, Sichuan University/West China School of Nursing, Sichuan University, Chengdu, China; ^2^West China School of Nursing, Sichuan University/Department of Neurology, West China Hospital, Sichuan University, Chengdu, China

**Keywords:** Parkinson's disease, family-centered care, barrier, facilitator, scoping review

## Abstract

**Aims:**

This study aimed to identify and synthesize the barriers and facilitators to the implementation of family-centered care in Parkinson's disease (PD) and to provide a reference for evidence-based Parkinson's disease nursing practice.

**Methods:**

This scoping review follows the methodology framework proposed by Arksey and O'Malley. Four databases including PubMed, Web of Science, Embase, and Cochrane Library were searched. Barriers and facilitators were summarized based on the ecological family-centered model.

**Results:**

Through a comprehensive literature search, 35 studies were found for this scoping review. Barriers and facilitators to implementing family-centered care in PD included physiological factors, environmental factors, culturally based conflicts, living arrangements, education or skills training, group experiences, and individual and family consultations.

**Conclusion:**

Implementing family-centered care in Parkinson's disease is essential to providing comprehensive care that improves outcomes for both PD patients and their family members.

## 1. Introduction

As the second most common neurodegenerative disease, Parkinson's disease (PD) has shown an overall increasing trend in prevalence in 150 countries worldwide from 1990 to 2019 (1). Compared to 1990, the number of disability-adjusted life-years for PD increased by 128.86% for all ages, resulting in a rise in disease burden from 14th to 10th place (2). This enormous disease burden presents significant challenges to the recovery of patients with PD. As the disease progresses, the motor symptoms (e.g., resting tremor, bradykinesia, and rigidity) and non-motor symptoms (e.g., neuropsychiatric symptoms, sleep disorders, and cognitive impairment) of PD will gradually appear and worsen (3). According to a survey in the Asia-Pacific region, psychosocial support was the most common unmet care need for PD patients, followed by pain and other symptoms (4). For caregivers, sudden challenges can affect their physical, psychological, and social wellbeing. The top three unmet care needs for caregivers were emotional support, instrumental support, and community support networks (5). These unmet care needs and challenges are particularly evident in settings where there is a lack of guidance from healthcare professionals.

Family-centered care has been widely used in pediatric and adult settings for over a century and is defined as promoting patient health through a partnership between families and healthcare professionals. In family-centered care, patients and caregivers are treated with respect and dignity, and communication is characterized by mutual equality and shared decisions (6). The elements of a universal model of family-centered care include the following: (a) collaboration among patients, caregivers, and healthcare providers, (b) consideration of family context, (c) dedicated policies and procedures, and (d) disease-specific education (7).

Implementing family-centered care for patients with PD and their caregivers has several benefits. First, it can help to alleviate some of the burden and improve patient and caregiver outcomes. A family-centered program provided social psychological counseling eight times a week to patients with PD and their caregivers, which successfully reduced the caregiver burden and improved patients' cognitive and emotional function (8). Second, family-centered care can help to improve self-management skills and ensure patient safety. Recently, more and more programs are providing education and consultations for PD patients and their caregivers based on mobile health to improve the self-management of the disease (9–11). This method is particularly applicable in non-institutionalized communities and home scenarios. Finally, family-centered care encourages open communication between the healthcare team and the patient and their family. This can lead to a better understanding of the patient's needs and preferences, and ultimately to more effective collaboration (12).

For healthcare providers, implementing family-centered care is a complex and thorough process that requires a detailed intervention plan. A guideline in Canada recommends that patients with PD should have a comprehensive care plan negotiated by the individual, their family and caregivers, and all healthcare providers (13). This emphasizes the important role of family members in supporting PD patients and involving them in decision-making about their care.

The implementation of family-centered care in healthcare is a multifaceted process that encompasses various promoting factors and obstacles. However, the literature on these factors is fragmented and lacks clarity at present. Thus, this study aimed at conducting a scoping review of the evidence on family-centered care in PD, compiling intervention measures, and analyzing the facilitators and barriers to family-centered care. In doing so, this study may provide a basis for evidence-based nursing practice in family-centered care of Parkinson's disease.

## 2. Methods

### 2.1. Study design

This scoping review follows the methodology framework proposed by Arksey and O'Malley, which includes five steps (14): (a) identifying the research question, (b) identifying relevant studies, (c) selecting studies, (d) charting the data, and (e) collating, summarizing, and reporting the results. According to the methodology framework, we identified the primary research question through a preliminary search: What are the barriers and facilitators for healthcare providers to implement family-centered care in PD? In other words, this study focuses on the elements of family-centered care in PD and the participatory roles of healthcare providers. In addition, this study was reported following the Preferred Reporting Items for Systematic Reviews and Meta-Analyses statements for Reporting Scoping Reviews (PRISMA-ScR). A meta-analysis was not performed due to the heterogeneity of the interventions.

### 2.2. Theoretical model

The ecological family-centered model was utilized to analyze the barriers and facilitators for healthcare providers to implement family-centered care in PD. The seven elements of this model were first proposed by Johnson et al. and included physiological factors, environmental factors, culturally-based conflicts, living arrangements, education or skills training, group experiences, and individual or family counseling (15). Subsequently, Monahan expanded the connotation of the model and applied it to family-centered care in dementia (16). In Monahan's opinion, the first three assessment factors (physiological factors, environmental factors, culturally-based conflicts, and living arrangements) could inform the rest of the interventions (skills training, group experiences, and individual or family counseling).

### 2.3. Search strategy

Four databases were comprehensively searched, namely PubMed, Web of Science, Embase, and Cochrane Library, from inception to 14 March 2023. Searches were limited to the English language. The search strategy involved two main components consisting of MeSH terms and keywords: (a) disease-related terms (e.g., Parkinsonian disorders, or Parkinson's disease) and (b) terms related to family-centered care (e.g., family support, family nursing, family therapy, caregiver, and carer). The complete search strategy is shown in Supplementary Table 1.

### 2.4. Eligibility criteria

Studies were included if they met the following criteria: (a) definition: family-centered care was defined as any interventions for patients with PD and their families; family-centered care programs were mainly developed by healthcare providers; patients with PD, caregivers, and healthcare providers were involved in promoting patient safety; (b) context: family-centered care was carried out in non-institutionalized settings, including homes, communities, and outpatient settings; and (c) study design: mixed methods study, clinical trials (including randomized and non-randomized controlled trials, pre-post studies, pilot studies, and case studies), and qualitative studies to evaluate the effects of family-centered care.

Studies were excluded if they met the following criteria: (a) they provided care only to patients with PD or caregivers; (b) they consisted of observational studies, such as case–control, cross-sectional, and cohort studies; (c) they consisted of protocols, reviews, editorials, comments, and conference abstracts; and, finally (d) they were not published in English.

### 2.5. Study selection and data charting

All search results were imported into Endnote X9 to remove duplicate results. Two reviewers screened the literature for titles and abstracts. Any discrepancies were resolved by a third reviewer. Key information was extracted by two reviewers using a custom data extraction table. Elements extracted included author, year, study design, country, aims of the study, participant characteristics, roles of healthcare providers, and content of family-centered care.

## 3. Results

### 3.1. Search results and selection

**Figure 1:**
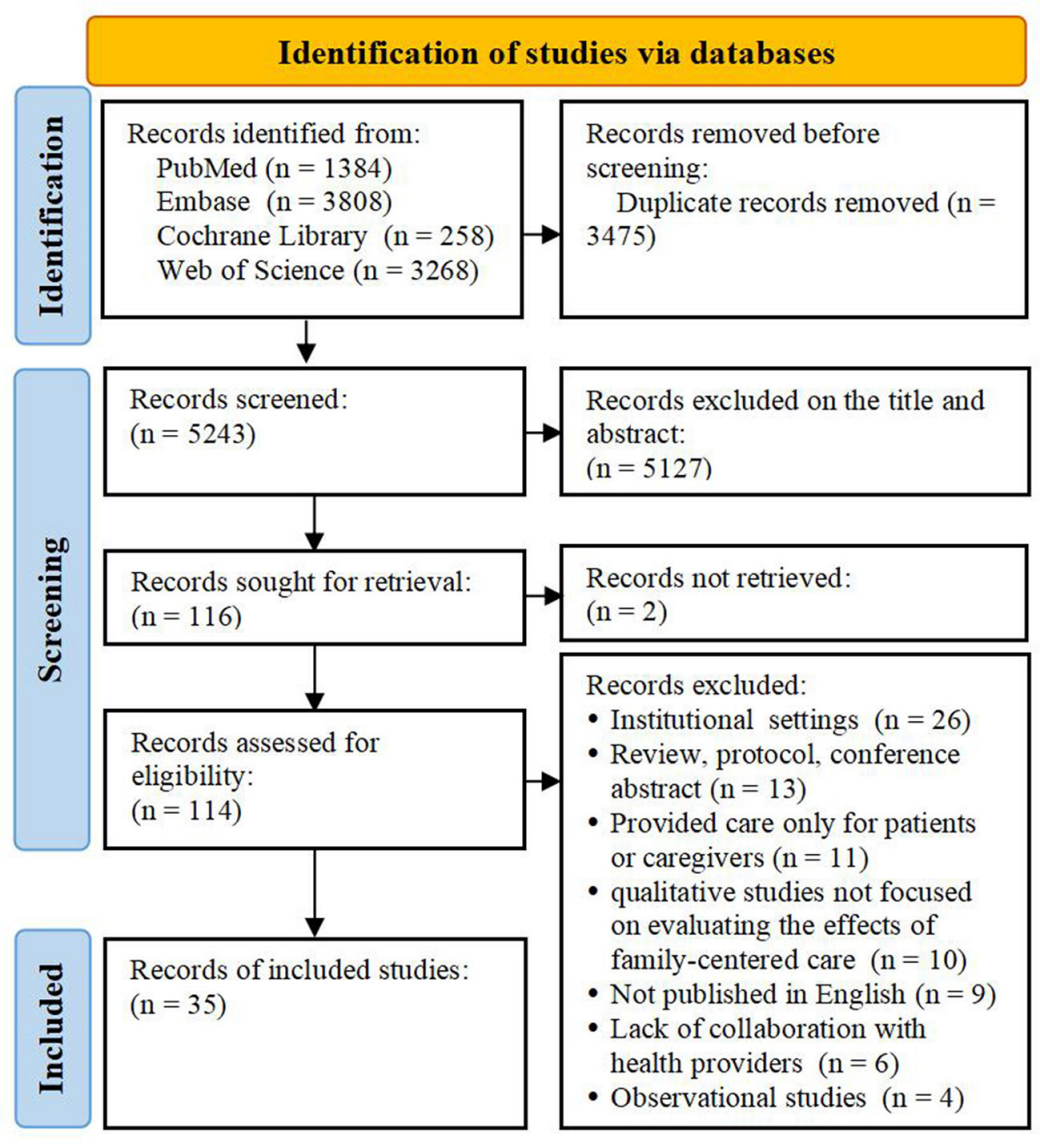
Flow diagram of study selection.

A total of 8,718 studies were retrieved. After duplicate records were removed, 5,423 studies were screened. Of these, 5,127 studies were excluded based on titles and abstracts. Although we attempted to contact the authors, the full texts of the two studies could not be obtained. According to the eligibility criteria, 79 studies were excluded for different reasons. Consequently, 35 studies were included in this scoping review. The flow diagram of the study selection is shown in Figure 1.

### 3.2. Study characteristics

Most of the studies were conducted in the United States (*n* = 15). Two studies were multicenter studies, one of which carried out a standardized psychological education program in seven European countries, and the other was conducted in the United States and Canada (17, 18). The rest of the studies were carried out in Australia (*n* = 5), the Netherlands (*n* = 4), England (*n* = 3), Canada (*n* = 2), Africa (*n* = 1), China (*n* = 1), Brazil (*n* = 1), and Sweden (*n* = 1). The study design of the selected studies included quantitative studies (*n* = 25) (8, 9, 17, 19–40), qualitative studies (*n* = 7) (10, 18, 41–45), and mixed method studies (*n* = 3) (11, 36, 46). Most of the studies were carried out in home settings (n = 18), followed by non-institutionalized communities (*n* = 8) and outpatient settings (*n* = 9). Study characteristics are detailed in Supplementary Table S2.

### 3.3. Distribution of research topics

By title and keyword identification, the research topics are summarized in Table 1. There were 31% of the research topics related to telemedicine (*n* = 5) (20, 24, 26, 28, 34) and cognitive-behavioral therapy (*n* = 6) (22, 24, 29, 30, 35, 40). Cognitive behavioral therapy has gradually become a family-centered research focus of Parkinson's since 2010, while telemedicine has gradually become a research hotspot since 2017 because of its technical reasons. Three studies used adherence therapy and care pathways to provide family-centered care for patients and caregivers, respectively (21, 32, 46). Sturkenboom focused on occupational therapy and published two studies in 2013 and 2016, respectively (36, 47). In addition, A' Campo focused on the psychosocial education program and published three RCTs (8, 17, 19). Although most studies adopted interdisciplinary cooperation, only two studies aimed to develop interdisciplinary programs (25, 39).

**Table 1:** Distribution of research topics.

**Theme**	***n***	**%**
Cognitive behavioral therapy	6	17.1
Telemedicine	5	14.3
Support group	4	11.4
Self-management	4	11.4
Palliative care	4	11.4
Psychosocial education	3	8.6
Interdisciplinary program	2	5.7
Physical exercise	2	5.7
Occupational therapy	2	5.7
Sleep therapy	1	2.9
Care pathway	1	2.9
Adherence therapy	1	2.9

### 3.4. Roles of healthcare providers involved in family-centered care

The overall role of healthcare providers was to develop a program of care for patients with PD, and their caregivers to address physical and psychological problems. In most of the studies, healthcare providers played the roles of planners, implementers, supervisors, and managers at the same time. Multidisciplinary teams were formed in eight studies (18, 25, 27, 28, 31, 39, 43, 44). Team members included physical, occupational, and speech therapists, movement disorders neurologists, movement disorders fellows, nurses, social workers, researchers, chaplains, graphic designers, and information technology experts. The roles of healthcare providers are outlined in Supplementary Table S2.

### 3.5. Barriers and facilitators to implementing family-centered care in PD

#### 3.5.1. Physiological and environmental factors

**Figure 2:**
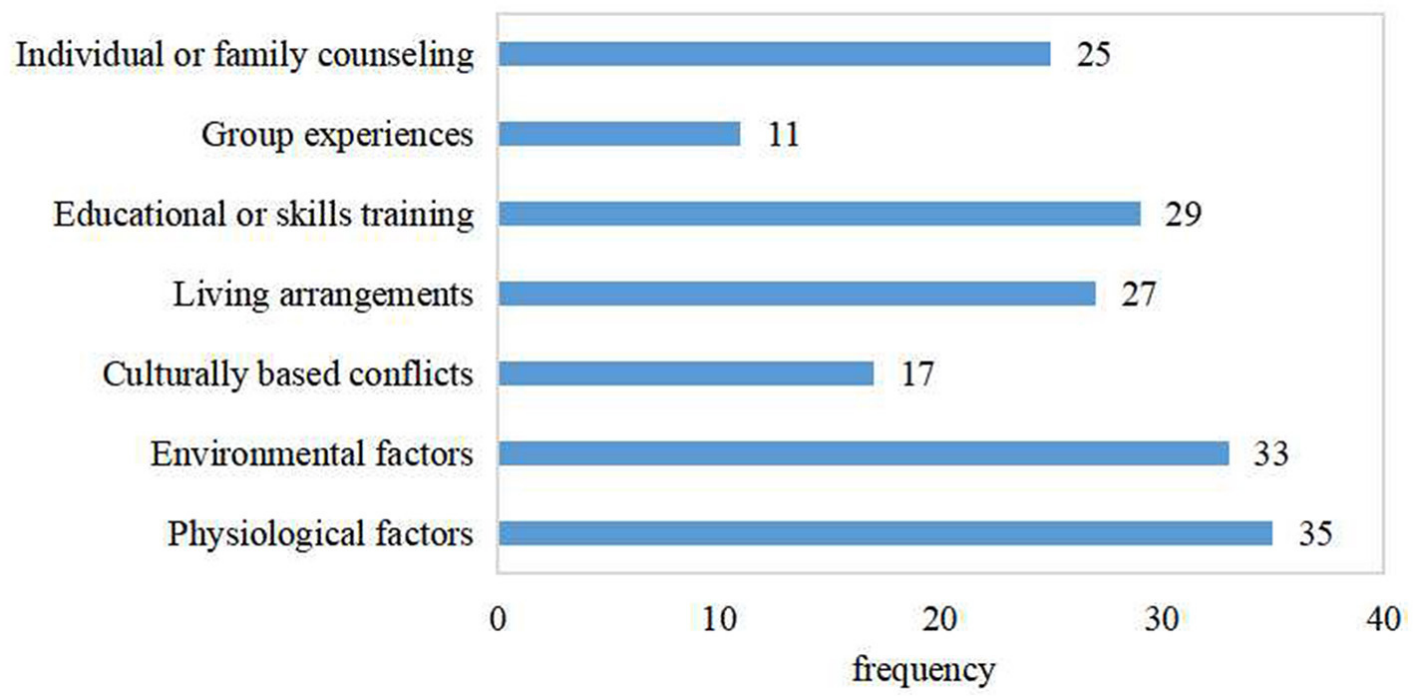
Elements of family-centered care in Parkinson's disease.

All 35 studies evaluated the physiological factors of PD from different aspects, which could be the facilitators for healthcare providers to implement family-centered care for Parkinson's disease. Only two studies did not pay enough attention to environmental assessment (21, 37). While most studies tended to develop holistic care plans for patients with PD at all stages, only a small number of case studies implemented case management according to the stages of PD (27, 30, 39). Providing information about advanced PD services to patients with relatively mild symptoms could make them feel anxious and uncomfortable (44, 46). Another barrier was that it may be difficult to fully assess physiological and environmental factors for remote intervention programs. Due to the lack of face-to-face consultation, it was difficult to assess the physiological changes before and after the intervention (20, 24). The elements of family-centered care in PD are illustrated in Figure 2.

#### 3.5.2. Culturally-based conflicts

The main barrier to this theme was the identification and handling of cultural conflicts. More than half of the studies did not assess the cultural background of patients and caregivers. The assessment of cultural conflict could make the intervention program more appropriate to the educational experience and cognitive level of patients and caregivers (8, 17, 19). For example, translating intervention manuals into different languages or involving linguists could facilitate communication between healthcare providers and families (8, 19).

#### 3.5.3. Living arrangements

Assessment of living arrangements and habits could facilitate the development of a personalized care plan. However, eight studies may have ignored the assessment of living arrangements. In the PD nursing process, studies tend to evaluate living arrangements on topics such as sleep therapy, cognitive behavioral therapy, palliative care, and physical exercise (18, 27, 29–31, 35, 38, 40, 44). In this regard, the main living arrangement has been the lack of a systematic and comprehensive living arrangement assessment scale.

#### 3.5.4. Education or skills training

Providing education and skills training could improve anxiety, depression, and caregiver burden in patients with PD (8, 17). In the process of education or skills training, controlling the difficulty of training and frequency by healthcare providers was an important barrier. Some psychological interventions took a long time to have a therapeutic effect (47). Frequent training may cause fatigue in patients with PD and their caregivers, while excessive training may result in poor intervention effects (46). Complex education or skills training may be confusing and difficult for patients and caregivers to understand (44). Another challenge in implementing education and skills training was to empower patients and caregivers, increase their engagement, and achieve self-management (9–11, 43, 47).

#### 3.5.5. Group experiences

Although only 11 studies focused on group discussions to share experiences, they all showed that patients and caregivers could gain experience and emotional support in different ways. Group discussions helped alleviate feelings of loneliness, isolation, and apathy among patients with PD and their caregivers while also providing them with peer support (8, 11, 27, 41, 43). During the group discussion, the main barrier was that some caregivers may consider some topics more sensitive, which could not be discussed in relatively large groups (43).

#### 3.5.6. Individual or family counseling

There were 25 studies on individual or family counseling. Patients with PD and their caregivers were willing to consult medical staff face-to-face. Nevertheless, the lack of consultation time and long intervals may hinder the intervention effects of family-centered care (27, 43, 44). Remote consultation gradually became a trend, which broke the limitations of time and space and made individual consultation more convenient (20, 23, 26, 28, 34). In addition, doctors and nurses played an active role in assisting in diagnosis and decision-making. However, there was a gap between physician recommendations and patient compliance when patients were in non-institutional settings (26).

## 4. Discussion

This scoping review pooled 35 studies to analyze the research hotspots of family-centered care and outlined the main interventions in PD. Based on the ecological model of family-centered care, this review explored the barriers and facilitators for healthcare providers to implement family-centered care in PD from seven domains. According to the results of the study, this review proposes the following suggestions for healthcare providers to implement family-centered care in PD.

### 4.1. Identifying family-centered care in PD as a terminology

Family-centered care in PD is a concept that has been discussed but has not yet been formally proposed as a terminology. In this scoping review, elements of family-centered care permeated all aspects of PD care in 35 studies. However, none of them treated family-centered care in PD as a complete terminology, which posed challenges to the literature search and screening. For example, in Dissanayaka's study, patients attended cognitive behavioral therapy sessions with their caregivers, and the intervention content was adjusted to the needs of patients with PD based on communication between healthcare providers and families (22). Although the explicit concept of family-centered care was not mentioned, Dissanayaka's study included key elements that align with family-centered care. Therefore, it is inappropriate to entirely dismiss and exclude this research. Owing to the lack of technical terminology, the concept of family-centered care delivered by healthcare providers is unclear, resulting in a gap in the description of family interventions. Thus, it is imperative to construct the concept of family-centered care. Moreover, identifying family-centered care in Parkinson's disease as a terminology will allow for the seamless integration of medical resources and facilitate the development of efficient intervention programs aimed at delivering high-quality nursing care to PD patients.

### 4.2. Involving advanced practice nurses in the provision of ongoing care

Advanced practice nurses in PD can diagnose and deal with individual and family health problems. Furthermore, they play an important role in chronic disease case management and teamwork (48). In this review, only one study involved advanced practice nurses in providing telephone calls and ongoing expert supervision to patients with PD and their caregivers (33). Ongoing care provided by advanced practice nurses for PD requires a clear role orientation and competency framework. The Parkinson's Disease Nurse Specialist Association, Parkinson's UK, and the Royal College of Nursing have jointly defined the career framework for advanced practice nurses in PD, which includes registered competence nurses, experienced specialist nurses, expert specialist nurses, and consultant nurses (49). In Germany, most PD nurses work in hospitals, resulting in a shortage of qualified specialists in outpatient and home settings (48). The involvement of advanced practice PD nurses will help to provide high-quality, continuous, and specialized care for patients with PD and their caregivers from hospital to community and home settings.

### 4.3. Encourage patients and their families to participate in self-management

Viewing the family as a whole and encouraging patients and caregivers to participate in self-management is necessary for the treatment and rehabilitation of PD. In this review, only 11.4% of the studies focused on the self-management of PD. It can be observed that patients with PD and their families still lack the awareness and methods of disease self-management. For example, the development of digital health technology systems and wearable body sensors, in addition to self-reporting of health status, has been shown to be effective in promoting self-management and rehabilitation in PD patients and their families (9, 43, 50).

### 4.4. Focus on family contexts and culturally-based conflicts

Patients with PD and their caregivers from different ethnicities and regions have different disease characteristics, cultural backgrounds, and lifestyles. Black patients with PD are more likely to develop dementia than white patients (OR: 1.33, 95% CI: 1.28–1.38) (51). Thus, they may have a greater need for cognitive behavioral training than white patients with PD. In this review, 77.1% of the studies analyzed the living arrangements of patients with PD, while 48.6% of the studies analyzed culturally-based conflicts. When focusing on cultural conflicts in patients with PD and their families, it is necessary to analyze the underlying causal mechanisms of such conflicts, such as patient values, socioeconomic conditions, and doctor–patient communication (52).

### 4.5. Rational use of telemedicine in a non-institutional setting

Telemedicine for PD provides remote treatment and rehabilitation for patients and caregivers through information and communication technologies. In this review, 14.3% of the studies preferred to provide telemedicine services to PD patients and their caregivers. Tremors, gait, and falls can be monitored and evaluated remotely through wearable devices and mobile applications (38). Medication and individual consultations can also be provided through online communities (10). Feasibility, cost and time savings, preferences of patients and doctors, and patient outcomes determine the feasibility of implementing telemedicine in PD. In addition, local restrictive regulations related to telemedicine should be strictly followed. For instance, telemedicine is illegal in South Korea (53). Overall, when healthcare professionals provide telemedicine services, patient privacy, information security, network speed, and communication equipment should be taken into account.

### 4.6. Limitations

Several limitations should be acknowledged in this scoping review. First, this study only included English-language literature, which may not be representative enough. Second, due to the heterogeneity of interventions and research methods, meta-analysis was not performed in this study. More studies based on different interventions of family-centered care in PD may help to address the heterogeneity. Finally, this study did not evaluate the quality of the included studies because it is not the content of the scoping review in the methodological framework proposed by Arksey and O'Malley (14).

## 5. Conclusion

Through a systematic literature search, this study summarized the key research issues of family-centered care in PD, explored the role of healthcare providers, and analyzed the barriers and facilitators to implementing family-centered care in PD. These include physiological factors, environmental factors, culturally-based conflicts, living arrangements, education or skills training, group experiences, and individual and family consultations. In the future, it will be necessary to further clarify the connotation of family-centered care in PD and attach importance to the role of healthcare providers in delivering ongoing care.

## Data availability statement

The original contributions presented in the study are included in the article/Supplementary material, further inquiries can be directed to the corresponding author.

## Author contributions

W-JS and YL designed the methodology. Y-JP, YL, and W-JS searched the literature and extracted the study characteristics. The manuscript was written by W-JS and revised by Y-JP. All authors contributed to the article and approved the submitted version.

## References

[1] Ou Z Pan J Tang S Duan D Yu D Nong H . Global trends in the incidence, prevalence, and years lived with disability of Parkinson's disease in 204 countries/territories from 1990 to 2019. Front Public Health. (2021) 9:776847. 10.3389/fpubh.2021.776847 34950630 PMC8688697

[2] Ding C Wu Y Chen X Chen Y Wu Z Lin Z . Global, regional, and national burden and attributable risk factors of neurological disorders: the global burden of disease study 1990–2019. Front Public Health. (2022) 10:952161. 10.3389/fpubh.2022.952161 36523572 PMC9745318

[3] Lewitt PA Chaudhuri KR. Unmet needs in Parkinson disease: motor and non-motor. Parkinsonism Relat Disord. (2020) 80 Suppl 1:S7–S12. 10.1016/j.parkreldis.2020.09.024 33349582

[4] Kwok JYY Huang T-W Tretriluxana J Auyeung M Chau PH Lin CC . Symptom burden and unmet support needs of patients with Parkinson's disease: a cross-sectional study in Asia-pacific regions. J Am Med Dir Assoc. (2021) 22:1255–64. 10.1016/j.jamda.2020.09.012 33268298

[5] Perrin PB Henry RS Donovan EK Cariello AN Lageman SK Villaseñor T . Parkinson's family needs and caregiver mental health: a cross-cultural comparison between Mexico and the United States. NeuroRehabilitation. (2019) 45:433–42. 10.3233/NRE-192894 31868689 PMC7025758

[6] Jolley J Shields L. The evolution of family-centered care. J Pediatr Nurs. (2009) 24:164–70. 10.1016/j.pedn.2008.03.010 19268238

[7] Kokorelias KM Gignac MAM Naglie G Cameron JI. Towards a universal model of family centered care: a scoping review. BMC Health Serv Res. (2019) 19:564. 10.1186/s12913-019-4394-5 31409347 PMC6693264

[8] A'Campo LEI Wekking EM Spliethoff-Kamminga NGA Le Cessie S Roos RAC. The benefits of a standardized patient education program for patients with Parkinson's disease and their caregivers. Parkinsonism Rel Disord. (2010) 16:89–95. 10.1016/j.parkreldis.2009.07.009 19674927

[9] Lyons KS Zajack A Greer M Chaimov H Dieckmann NF Carter JH. Benefits of a self-management program for the couple living with Parkinson's disease: a pilot study. J Appl Gerontol. (2021) 40:881–9. 10.1177/0733464820918136 32401118

[10] Nunes F Andersen T Fitzpatrick G. The agency of patients and carers in medical care and self-care technologies for interacting with doctors. Health Informatics J. (2019) 25:330–49. 10.1177/1460458217712054 28653552

[11] Pappa K Doty T Taff SD Kniepmann K Foster ER. Self-management program participation and social support in Parkinson's disease: mixed methods evaluation. Phys Occup Ther Geriatr. (2017) 35:81–98. 10.1080/02703181.2017.1288673 29203950 PMC5711466

[12] Pitts E Wylie K Loftus AM Cocks N. Communication strategies used by Parkinson's nurse specialists during healthcare interactions: a qualitative descriptive study. J Adv Nurs. (2022) 78:1773–86. 10.1111/jan.15196 35285973 PMC9313789

[13] Grimes D Fitzpatrick M Gordon J Miyasaki J Fon EA Schlossmacher M . Canadian guideline for Parkinson disease. CMAJ. (2019) 191:E989–E1004. 10.1503/cmaj.181504 31501181 PMC6733687

[14] Arksey H O'Malley L. Scoping studies: towards a methodological framework. Int J Soc Res Methodol. (2005) 8:19–32. 10.1080/1364557032000119616

[15] Johnson HC. Emerging concerns in family therapy. Soc Work. (1986) 31:299–306. 10.1093/sw/31.4.299

[16] Monahan DJ. Assessment of dementia patients and their families: an ecological-family-centered approach. Health Soc Work. (1993) 18:123–31. 10.1093/hsw/18.2.123 8288140

[17] A'Campo LE Spliethoff-Kamminga NG Macht M EduPark C Roos RA. Caregiver education in Parkinson's disease: formative evaluation of a standardized program in seven European countries. Qual Life Res Int J Qual Life Aspects Treat Care Rehabil. (2010) 19:55–64. 10.1007/s11136-009-9559-y 19946755 PMC2804793

[18] Jordan SR Kluger B Ayele R Brungardt A Hall A Jones J . Optimizing future planning in Parkinson disease: suggestions for a comprehensive roadmap from patients and care partners. Ann Palliat Med. (2020) 9:S63–74. 10.21037/apm.2019.09.10 32036671 PMC7408313

[19] A'Campo LEI Wekking EM Spliethoff-Kamminga NGA Stijnen T Roos RAC. Treatment effect modifiers for the patient education programme for Parkinson's disease. Int J Clin Pract. (2012) 66:77–83. 10.1111/j.1742-1241.2011.02791.x 22171907

[20] Beck CA Beran DB Biglan KM Boyd CM Dorsey ER Schmidt PN . National randomized controlled trial of virtual house calls for Parkinson disease. Neurology. (2017) 89:1152–61. 10.1212/WNL.0000000000004357 28814455 PMC5595275

[21] Daley DJ Deane KHO Gray RJ Clark AB Pfeil M Sabanathan K . Adherence therapy improves medication adherence and quality of life in people with Parkinson's disease: a randomised controlled trial. Int J Clin Pract. (2014) 3:2439. 10.1111/ijcp.12439 24750544

[22] Dissanayaka NNW Pye D Mitchell LK Byrne GJ O'Sullivan JD Marsh R . Cognitive behavior therapy for anxiety in Parkinson's disease: outcomes for patients and caregivers. Clin Gerontol. (2017) 40:159–71. 10.1080/07317115.2016.1240131 28452666

[23] Dobkin RD Interian A Durland JL Gara MA Menza MA. Personalized telemedicine for depression in Parkinson's disease: a pilot trial. J Geriatr Psychiatry Neurol. (2018) 31:171–6. 10.1177/0891988718783274 29945467

[24] Dobkin RD Menza M Allen LA Tiu J Friedman J Bienfait KL . Telephone-based cognitive-behavioral therapy for depression in Parkinson's disease. J Geriatr Psychiatry Neurol. (2011) 24:206–14. 10.1177/0891988711422529 22228827 PMC3571630

[25] Fleisher J Barbosa W Sweeney MM Oyler SE Lemen AC Fazl A . Interdisciplinary home visits for individuals with advanced Parkinson's disease and related disorders. J Am Geriatr Soc. (2018) 66:1226–32. 10.1111/jgs.15337 29608779 PMC6105368

[26] Fleisher JE Suresh M Klostermann EC Lee J Hess SP Myrick E . IN-HOME-PD Caregivers: The effects of a combined home visit and peer mentoring intervention for caregivers of homebound individuals with advanced Parkinson's disease. Parkinsonism Relat Disord. (2023) 106:14. 10.1016/j.parkreldis.2022.11.014 36446676 PMC9825655

[27] Fleisher JE Klostermann EC Hess SP Lee J Myrick E Chodosh J. Interdisciplinary palliative care for people with advanced Parkinson's disease: a view from the home. Ann Palliat Med. (2020) 9:S80–9. 10.21037/apm.2019.09.12 31735037 PMC7341729

[28] Gao S Hou Y Ma R Kaudimba KK Jin L Wang H . A Novel Management platform based on personalized home care pathways for medicine management and rehabilitation of persons with Parkinson's disease-requirements and implementation plan of the care-PD program. Front Neurol. (2021) 12:208. 10.3389/fneur.2021.672208 34113314 PMC8186830

[29] Giguere-Rancourt A Plourde M Racine E Couture M Langlois M Dupre N . Goal management training and psychoeducation / mindfulness for treatment of executive dysfunction in Parkinson's disease: a feasibility pilot trial. PLoS ONE. (2022) 17:108. 10.1371/journal.pone.0263108 35180229 PMC8856541

[30] Giguère-Rancourt A Plourde M Doiron M Langlois M Dupré N Simard M. Goal management training^®^ home-based approach for mild cognitive impairment in Parkinson's disease: a multiple baseline case report. Neurocase. (2018) 24:276–86. 10.1080/13554794.2019.1583345 30821637

[31] Kluger BM Miyasaki J Katz M Galifianakis N Hall K Pantilat S . Comparison of integrated outpatient palliative care with standard care in patients with parkinson disease and related disorders: a randomized clinical trial. JAMA Neurol. (2020) 77:551–60. 10.1001/jamaneurol.2019.4992 32040141 PMC7042842

[32] Leroi I Baker P Kehoe P Daniel E Byrne EJ A. pilot randomized controlled trial of sleep therapy in Parkinson's disease: effect on patients and caregivers. Int J Geriatr Psychiatry. (2010) 25:1073–9. 10.1002/gps.2472 20157905

[33] Pretzer-Aboff I Galik E Resnick B. Feasibility and impact of a function focused care intervention for Parkinson's disease in the community. Nurs Res. (2011) 60:276–83. 10.1097/NNR.0b013e318221bb0f 21691241

[34] Schindler N Bay AA Perkins MM Jackson J Ni L Pothineni S . Remote and in-person research education for people with Parkinson's disease and their care partners. Fam Syst Health. (2022) 4:684. 10.1037/fsh0000684 35737554 PMC9826735

[35] Seritan AL Iosif A-M Prakash P Wang SS Eisendrath S. Online mindfulness-based cognitive therapy for people with Parkinson's disease and their caregivers: a pilot study. J Technol Behav Sci. (2022) 7:381–95. 10.1007/s41347-022-00261-7 35527798 PMC9059916

[36] Sturkenboom IH Graff MJ Borm GF Veenhuizen Y Bloem BR Munneke M . The impact of occupational therapy in Parkinson's disease: a randomized controlled feasibility study. Clin Rehabil. (2013) 27:99–112. 10.1177/0269215512448382 22811447

[37] Tamplin J Morris ME Marigliani C Baker FA Noffs G Vogel AP. ParkinSong: outcomes of a 12-month controlled trial of therapeutic singing groups in Parkinson's disease. J Parkinson's Dis. (2020) 10:1217–30. 10.3233/JPD-191838 32538865

[38] Torriani-Pasin C Domingues VL de Freitas TB da Silva TA Caldeira MF Alcantaro Junior RP . Adherence rate, barriers to attend, safety and overall experience of a physical exercise program via telemonitoring during COVID-19 pandemic for individuals with Parkinson's disease: a feasibility study. Physioth Res Int. (2022) 27:1959. 10.1002/pri.1959 35633094 PMC9348085

[39] Vickers LF O'Neill CM. An interdisciplinary home healthcare program for patients with Parkinson's disease. Rehabilitation nursing. J Assoc Rehabil Nurses. (1998) 23:286–99. 10223029 10.1002/j.2048-7940.1998.tb01806.x

[40] Wuthrich VM Rapee RM. Telephone-delivered cognitive behavioural therapy for treating symptoms of anxiety and depression in Parkinson's disease: a pilot trial. Clin Gerontol. (2019) 42:444–53. 10.1080/07317115.2019.1580811 30821649

[41] Abell RV Baird AD Chalmers KA. Group singing and health-related quality of life in Parkinson's disease. Health Psychol. (2017) 36:55–64. 10.1037/hea0000412 27584976

[42] Fothergill-Misbah N Moffatt S Mwithiga H Hampshire K Walker R. The role of support groups in the management of Parkinson's disease in Kenya: sociality, information and legitimacy. Glob Public Health. (2022) 17:1773–83. 10.1080/17441692.2021.1954227 34255606

[43] Hellqvisti C Dizdar N Hagell P Bertero C Sund-Levander M. Improving self-management for persons with Parkinson's disease through education focusing on management of daily life: patients' and relatives' experience of the Swedish National Parkinson School. J Clin Nurs. (2018) 27:3719–28. 10.1111/jocn.14522 29782061

[44] Lum HD Jordan SR Brungardt A Ayele R Katz M Miyasaki JM . Framing advance care planning in Parkinson disease: patient and care partner perspectives. Neurology. (2019) 92:e2571–9. 10.1212/WNL.0000000000007552 31028124 PMC6556088

[45] Prieto L Norris ML Columna L. “Keep Moving”: Experiences of people with Parkinson's and their care partners in a dance class. Adap Physical Act Quart: APAQ. (2021) 38:307–28. 10.1123/apaq.2019-0125 33596544

[46] Holloway M. Traversing the network: a user-led Care Pathway approach to the management of Parkinson's disease in the community. Health Soc Care Commun. (2006) 14:63–73. 10.1111/j.1365-2524.2005.00600.x 16324188

[47] Sturkenboom IH Nijhuis-van der Sanden MW Graff MJ. A process evaluation of a home-based occupational therapy intervention for Parkinson's patients and their caregivers performed alongside a randomized controlled trial. Clin Rehabil. (2016) 30:1186–1199. 26672997 10.1177/0269215515622038

[48] Prell T Siebecker F Lorrain M Tönges L Warnecke T Klucken J . Specialized staff for the care of people with parkinson's disease in germany: an overview. J Clin Med. (2020) 9:2581. 10.3390/jcm9082581 32784969 PMC7463847

[49] Parkinson's Disease Nurse Specialist Association,. Competencies: A Competency Framework For Nurses Working in Parkinson's Disease Management. (2023). Available online at: https://www.parkinsons.org.uk/sites/default/files/2017-12/competency_framework_for_parkinsons_nurses_2016.pdf (accessed April 10, 2023).

[50] Debelle H Packer E Beales E Bailey HGB Mc Ardle R Brown P . Feasibility and usability of a digital health technology system to monitor mobility and assess medication adherence in mild-to-moderate Parkinson's disease. Front Neurol. (2023) 14:1111260. 10.3389/fneur.2023.1111260 37006505 PMC10050691

[51] Ben-Joseph A Marshall CR Lees AJ Noyce AJ. Ethnic variation in the manifestation of Parkinson's disease: a narrative review. JPD. (2020) 10:31–45. 10.3233/JPD-191763 31868680 PMC7029316

[52] Smith ER Perrin PB Tyler CM Lageman SK Villaseñor T. Cross-cultural differences in Parkinson's disease caregiving and burden between the United States and Mexico. Brain Behav. (2020) 10:1753. 10.1002/brb3.1753 32683797 PMC7507106

[53] Shalash A Spindler M Cubo E. Global perspective on telemedicine for Parkinson's disease. J Parkinsons Dis. (2021) 11:S11–8. 10.3233/JPD-202411 33579872 PMC8385495

