# Supplementary material for: Barriers and facilitators for healthcare providers to implement family-centered care in Parkinson's disease: a scoping review
Source: Front Neurol. 2023 Aug 25;14:1231654. doi: 10.3389/fneur.2023.1231654 (PMC10486989; doi:10.3389/fneur.2023.1231654)
Supplement: Supplementary file 1 [file Data_Sheet_1.doc]

Supplementary Material

### 1.1 Supplementary Table S1. Search strategy……………………………………………………2

### 1.2 Supplementary Table S2. Study characteristics………………………………………………5

### 1.3 Supplementary Table S3. PRISMA-ScR……………………………………………………23

# Supplementary Tables

### 1.1 Supplementary Table S1. Search strategy

Search Date: up to 2023/3/14

| **Database** | **No** | **Search strategy** | **Results** |
| --- | --- | --- | --- |
| PubMed | #1 | "Parkinsonian Disorders"[MeSH Terms] OR "Parkinson Disease"[MeSH Terms] | 1384 |
|  | #2 | "parkinson*"[Title/Abstract] |  |
|  | #3 | #1 OR #2 |  |
|  | #4 | "Caregivers"[MeSH Terms] OR "Family Support"[MeSH Terms] OR "Family Nursing"[MeSH Terms] OR "Family Therapy"[MeSH Terms] |  |
|  | #5 | "caregiver*"[Title/Abstract] OR "carer*"[Title/Abstract] OR "family-centered"[Title/Abstract] OR "family centred"[Title/Abstract] |  |
|  | #6 | #4 OR #5 |  |
|  | #7 | "review"[Publication Type] OR "systematic review"[Publication Type] OR "Meta-analysis"[Publication Type] OR "comment"[Title] OR "editorial"[Title] OR "protocol"[Title] |  |
|  | #8 | (#3 AND #6) NOT #7 |  |
| **Database** | **No** | **Search strategy** | **Results** |
| Embase | #1 | 'Parkinson disease'/exp OR 'parkinsonism'/exp | 3808 |
|  | #2 | (Parkinson*):ab,ti |  |
|  | #3 | #1 OR #2 |  |
|  | #4 | 'caregiver'/exp OR 'family centered care'/exp OR 'family therapy'/exp OR 'family support'/exp |  |
|  | #5 | caregiver*:ab,ti OR carer:ab,ti OR 'family-centered':ab,ti OR 'family- centred':ab,ti OR 'family centered':ab,ti |  |
|  | #6 | #4 OR #5 |  |
|  | #7 | reveiw:it OR 'systematic review':it OR 'meta analysis':it OR comment:it OR editorial:it OR protocol:it |  |
|  | #8 | #3 AND #6 |  |
|  | #9 | #8 NOT #7 |  |
| **Database** | **No** | **Search strategy** | **Results** |
| Cochrane Library | #1 | MeSH descriptor: [Parkinson Disease] explode all trees | 258 |
|  | #2 | MeSH descriptor: [Parkinsonian Disorders] explode all trees |  |
|  | #3 | (Parkinson*):ti,ab,kw |  |
|  | #4 | MeSH descriptor: [Family Nursing] explode all trees |  |
|  | #5 | MeSH descriptor: [Family Support] explode all trees |  |
|  | #6 | MeSH descriptor: [Family] explode all trees |  |
|  | #7 | MeSH descriptor: [Caregivers] explode all trees |  |
|  | #8 | (caregiver* OR family-centered OR family centered OR carer*):ti,ab,kw |  |
|  | #9 | (reveiw OR systematic review OR Meta-analysis OR comment OR editorial OR protocol):pt |  |
|  | #10 | #1 OR #2 OR #3 |  |
|  | #11 | #4 OR #5 OR #6 OR #7 OR #8 |  |
|  | #12 | (#10 AND #11) NOT #9 |  |
| **Database** | **No** | **Search strategy** | **Results** |
| Web of Science | #1 | TS=(Parkinson's Disease) OR TS=(Parkinson*) OR TS=(Parkinson disease) | 3268 |
|  | #2 | TS=(family centered) OR TS=(family-centered) OR TS=(family nurisng) OR TS=(family care) OR TS=(Caregiver*) OR TS=(carer) |  |
|  | #3 | ((((TI=(systematic review)) OR TI=(meta-analysis)) OR TI=(editorial)) OR TI=(protocol)) OR TI=(comment) |  |
|  | #4 | (#1 AND #2) NOT #3 |  |
| **Total** |  |  | **8718** |

### 1.2 Supplementary Table S2. Study characteristics

| **Study**  1. Author  2. Year  3. Study design  4. Country | **Aim** | **Participants characteristics** | **Roles of healthcare providers** | **Contents of family-centered care** |
| --- | --- | --- | --- | --- |
| 1. Vickers, et al.  2. 1998  3. Case study  4. USA | This article describes the Affiliated Community Visiting Nurse Association, Inc (ACVNA) program, which has become a national model for other home healthcare agencies hoping to start or expand Parkinson's disease programs, and presents a case study. | 1. Patients (n=1, age: 72) 2. Caregivers (n=1) 3. Setting: home | The PD team consists of physical, occupational, and speech therapists, as well as home health aides with special training in reinforcing motor replanning and other compensation techniques. | Team members implement the patient's plan of care, teach the patient exercises and compensation techniques, recommend adaptive equipment to enable the patient to maintain independence and safety, teach the patient and caregivers about the signs and symptoms of disease progression, the medication regimen, and coping skills, and recommend additional therapy or counseling if needed. |
| 1. Holloway, et al. 2. 2006 3. Mixed method study 4. UK | To develop and implement a Care Pathway framework for people with Parkinson’s disease and their caregivers, to facilitate more comprehensive and integrated health and social care, with a streamlining of the transfer of core information around the system. | 1. Patients and caregivers (n=22) 2. Settings: home | The Care Pathway tools, comprising a local information pack, a Problems/Needs form, a Clinic Summary and a service record sheet, were designed by a working party consisting of service providers, a service user and carer and the researchers. | Phase 1. Establish the arrangements for implementing the complete Care Pathway framework.  Phase 2. Participants were asked to use the Care Pathway forms for approximately 12 months from recruitment to last clinic visit.  Phase 3. semi-structured interviews were conducted with 22 participants after the end of the 12month intervention period. |
| **Study**  1. Author  2. Year  3. Study design  4. Country | **Aim** | **Participants characteristics** | **Roles of healthcare providers** | **Contents of family-centered care** |
| 1. A’ Campo, Spliethoff-Kamminga, et al. 2. 2010 3. RCT 4. Estonia, Finland, UK, Germany, Italy, Spain, The Netherlands | The formative evaluation of a standardized psychosocial education program for patients with Parkinson’s disease and their caregivers. | 1. Patients(n=151, age: 64.4±9.2) 2. Caregivers (n=137, age:62.2±11.3) 3. Setting: outpatient | Trainers were professional group leaders, mostly psychologists, who were knowledgeable about patient education and the psychosocial problems of PD. | The program consisted of eight weekly sessions of ninety minutes. Groups consisted of 4–7 participants. The program is based on principles of the cognitive-behavioral therapy. Interventions like systematic relaxation training, cognitive restructuring, situational behavioral analysis and training in social skills were included. |
| 1. A’ Campo, Wekking, et al. 2. 2010 3. RCT 4. The Netherlands | Aim at improving the health-related quality of life (Hr-Qol) of patients with Parkinson's disease (PD) and caregivers. | 1. Patients(n=61) 2. Caregivers (n=44) 3. Setting: outpatient | Trainers were professional group leaders, mostly psychologists, who were knowledgeable about patient education and the psychosocial problems of PD. | Patient Education Program Parkinson (PEPP) includes eight theme: information, self-monitoring, health promotion, stress management, management of anxiety and depression (patients)/caregiver's challenge, social competence, social support, evaluation |
| 1. Leroi, et al. 2. 2010 3. RCT 4. UK | To investigate the efficacy, tolerability and feasibility of a multi-component sleep therapy intervention versus basic sleep hygiene education in PD patients with sleep disturbances and their live-in carers. | 1. Patients(n=115) 2. Caregivers (n=15) 3. Setting: outpatient | The Multi-component sleep therapy intervention (MST) was undertaken by a mental health nurse with experience in education about health and lifestyle changes. | The MST included two components. The behavioural therapy involved suggesting to the PD sufferer and their carer that they attempt to eliminate the sleep-incompatible behaviours to regulate the sleep-wake cycle, and to consolidate sleep over a shorter period of time spent in bed. The educational component involved the effects of diet, exercise, substance use, light, noise and temperature. |
| 1. Dobkin, et al. 2. 2010 3. RCT 4. USA | To examine the feasibility and effect of telephone-based cognitive-behavioral therapy for depression in Parkinson’s disease | 1. Patients(n=21, age: 65.86±9.38 ) 2. Caregivers (n=21) 3. Setting: home | Phone-based CBT was provided by the first author, as well as 1 postdoctoral fellow, and 2 masters-level clinicians with background and training in CBT and health psychology. | Patients with Parkinson’s diseases received 10 sessions (60-90 minutes each) of CBT which was delivered over the phone and incorporated behavioral activation, recommendations to gradually increase exercise, thought monitoring and restructuring, relaxation training, worry control and sleep hygiene. Caregivers received up to four telephone-based educational sessions (30-60 minutes each). |
| 1. Pretzer-Aboff, et al. 2. 2011 3. Pre-post repeated measure study 4. USA | To test the feasibility and impact of function-focused care for PD (FFC-PD) developed to optimize function and physical activity among people with PD who live in the community. | 1. Patients(n=21, age: 71.7±8.5) 2. Caregivers 3. Setting: community | Advanced practice nurse provided telephone calls and ongoing expert oversight for the dyad. | The FFC-PD intervention involves four steps: implementing a function-focused philosophy of care, setting FFC goals, mentoring and motivating, expert oversight. |
| 1. A'Campo, et al. 2. 2012 3. Second analysis of RCT 4. The Netherlands | To search for treatment effect modifiers using secondary analyses of data from our randomized controlled trial in which PD patients as well as the caregivers participated in the program. | 1. Patients(n=65) 2. Caregivers (n=47) 3. Setting: outpatient | Trainers were professional group leaders, mostly psychologists, who were knowledgeable about patient education and the psychosocial problems of PD. | The programme’s key element includes techniques from the cognitive behavioural therapy, like cognitive restructuring, systematic relaxation training, situational behavioural analysis and training in social skills. |
| 1. Sturkenboom, et al. 2. 2013 3. RCT 4. The Netherlands | To investigate the efficacy, tolerability and feasibility of a multi-component sleep therapy intervention versus basic sleep hygiene education in PD patients with sleep disturbances and their live-in carers. | 1. Patients(n=43, age: 61.8±14.3) 2. Caregivers (n=43, age: 67.3±11.2) 3. Setting: home | The therapist formulated the treatment plan based on family information and additional information from the therapist’s own diagnostic phase. | The intervention was delivered at home for 10 weeks within three months while the number of sessions could vary depending on complexity of goals, with a maximum of 16 sessions (45-60 minutes). |
| 1. Daley, et al. 2. 2014 3. RCT 4. UK | To examine where there would be a statistically significant difference in medication adherence and QoL in PD patients who received adherence therapy (AT) . | 1. Patients(n=76, age: 71.9±8.9 ) 2. Caregivers (n=48) 3. Setting: home | AT was implemented under the supervision of a senior therapist/investigator. | AT focused on four areas:  • Assessment: medication review, exploring attitudes towards medication, side effects and agenda setting.  • Problem-solving: practical issues leading to non-adherence.  • Medication timeline: reflection on experience with medication.  • Medication ambivalence/beliefs and concerns: discussing pros & cons to taking/not taking medication. |
| 1. Sturkenboom, et al. 2. 2016 3. mixed method study 4. The Netherlands | To evaluate fidelity, treatment enactment and the experiences of an occupational therapy intervention in Parkinson’s disease, to identify factors that affect intervention delivery and benefits. | 1. Patients (n=123) 2. Caregivers (n=105) 3. Setting: home | As members of ParkinsonNet, all therapists received at least 3 days training on treating patients with Parkinson’s disease. | The intervention was a 10-week home-based, client-centered occupational therapy intervention according to the Dutch guidelines. The maximum contact time was 16 hours. The intervention focused on ‘the person’ (coaching and strategy training of recipient), the activity (adaptations of activities and daily routines) and the ‘environment’ (e.g. assistive devices, layout, and support). |
| 1. Abell, et al. 2. 2017 3. Qualitative study 4. Australia | To investigate the effects of group singing on health-related quality of life for people diagnosed with PD | 1. Patients and caregivers (n=11, mean age: 70.6) 2. Setting: Community | The choir is an á cappella group led by a qualified choir maestro who holds an Advanced Diploma in The-ater and Music . | Choir members meet for afternoon tea before the choir session proper. Choir sessions commence with a warm-up (approximately 20 min) which consists of deep breathing, stretching, and vocal exercises led by the choir maestro. Singing takes place for the following 90 min. The choir maestro selects the musical repertoire in consultation with the choir members. |
| 1. Beck, et al. 2. 2017 3. RCT 4. USA | To determine whether providing remote neurologic care into the homes of people with Parkinson disease (PD) is feasible, beneficial, and valuable. | 1. Patients(n=195, age: 66.4±8.1) 2. Caregivers 3. Setting: home | Neurologists provided virtual visits. | Individuals randomized to the intervention received up to 4 virtual visits over 12 months from a neurologist. The patient and physician determined the specific content and frequency of each visit, but the format generally included a medical history; a PD-specific examination, including assessment of tremor and gait; time to address patients' concerns; and recommendations. |
| 1. Dissanayaka, et al. 2. 2017 3. Pilot study 4. Australia | To evaluated the effects of cognitive behaviour therapy on patients with Parkinson’s disease and their caregivers. | 1. Patients (n=17, age: 66.6±8.0) 2. Caregivers (n=15, age: 63.4±9.7) 3. Setting: outpatient | All therapists were postgraduate students and registered psychologists of the clinical psychology program. Students were  supervised by experienced registered doctoral-level  psychologists. | Six intervention sessions covered the following domains: (i) psychoeducation; (ii) symptom monitoring; (iii) calming techniques including deep breathing; (iv) progressive muscle relaxation and imagery; (v) sleep hygiene; and (vi) self-management and relapse prevention planning. At each session, participants were given a take home handout to be completed prior to attending the subsequent session. |
| 1. Pappa, et al. 2. 2017 3. mixed method study 4. USA | To explore the potential influence of the Stanford Chronic Disease Self-Management Program on social support in Parkinson disease. | 1. Patients(n=46, age: 68.0±6.9) 2. Caregivers (n=6, age: 69.2 ±6.7) 3. Setting: community | Classes were led by two trained facilitators who guided participants through general information and activities that promote management of personal health using a scripted leader’s manual. | Each class series consists of six 2.5 hour classes held weekly in a community setting. Each participant received a copy of the companion book, Living a Healthy Life with Chronic Conditions, 4th Edition, which provides  to supplement class material. Participants were required to attend at least four of the six classes to be included in the study. |
| 1. Dobkin, et al. 2. 2018 3. Pilot study 4. USA | The purpose of this pilot project was to evaluate the feasibility and impact of a personalized cognitive-behavioral telemedicine program for depression in Parkinson’s disease. | 1. Patients (n=34, age: 62.6±9.5) 2. Caregivers 3. Setting: home | Treatment was provided by the first author and 2 master-level clinicians, working under her supervision. All study therapists were trained in the psychiatric complications of PD, prior to treating individual participants. | Treatment modules targeted behavioral activation, cognitive restructuring, anxiety management, and sleep hygiene. The intervention also incorporated a variety of cognitive–behavioral techniques for addressing the negative moods (shame, stigma, and anxiety) that commonly develop in response to PD symptoms (eg, falls, tremor, and wavering voice) as well as changes in self-image that can occur within PD. |
| 1. Fleisher, et al. 2. 2018 3. Pilot study 4. USA | To implement the interdisciplinary home visit program and provide continuity of expert neurologic care to homebound individuals with advanced PD and related disorders | 1. Patients(n=85, age: 72.5-84.8) 2. Caregivers 3. Setting: home | The Advanced Parkinson’s Disease (HVP) team consists of one movement disorders neurologist, one movement disorders fellow, one nurse, and one social worker/program coordinator, each with training in movement disorders and geriatric experience. | Social worker: review healthcare utilization & in-home services, psychosocial needs assessment of patient & caregiver, goals of care, discussion & education, supportive counseling. Registered nurse: review falls, vitals and orthostatics, real-time medication reconciliation, home safety review, aware in care kit education. Movement disorder specialist: Interim history, Physical examination, Assessment, plan, education, counseling |
| 1. Giguère-Rancourt, et al. 2. 2018 3. Case study 4. Canada | To assess the tolerability and safety of Adapted-Goal Management Training (GMT) administered to PD mild cognitive impairment patients and involve caregiver in cognitive training. | 1. Patients (n=1, age: 72) 2. Caregivers (n=1) 3. Setting: home | All assessments and interventions were performed by a Senior PhD Candidate under the supervision of a registered neuropsychologist and were conducted at the participant’s home. | GMT is a well-validated cognitive training developed to improve executive functions. GMT comprises self-instruction strategies, self-monitoring exercises, cognitive techniques, mindfulness exercises and assignment between sessions. It helps patients to raise awareness of deficits and improve cognitive control in goal-directed behaviors. |
| 1. Hellqvisti, et al. 2. 2018 3. Qualitative study 4. Sweden | To identify and describe experiences valuable for managing daily life after participation in the NPS self-management intervention and explore the applicability of the Self-and family management framework by Grey and colleagues for persons with Parkinson's disease and their relatives. | 1. Patients(n=25) 2. Caregivers (n=17 ) 3. Setting: outpatient | The National Parkinson School (NPS) was developed through collaboration among healthcare providers, researchers, the pharmaceutical industry and patient organizations. | The NPS consists of seven sessions where topics concerning PD and life with chronic disease are in focus through educational sessions, group discussions and home assignments, for example managing stress in daily life. |
| 1. Lum, et al. 2. 2019 3. Qualitative study 4. USA | To elicit perspectives from patients and care partners on Advance care planning (ACP) to inform a patient- and care partner-centered framework for PD clinical care and research. | 1. Patients (n=30, age: 66±8.0) 2. Caregivers (n=30, age: 68±7) 3. Setting: outpatient | Primary care provider and neurologist participated in an interdisciplinary team. | This qualitative descriptive study leverages a large, multisite, randomized clinical trial of interdisciplinary outpatient neuropalliative care compared to standard neurologic care for individuals with PD and care partners. |
| 1. Nunes, et al. 2. 2019 3. Qualitative study 4. USA | Explore the interactions patients and carers living with Parkinson’s have with their neurologists. | 1. Patients 2. Caregivers 3. Setting: online community | Interact with patients and caregivers. | There were five different interactions: (1) making explicit issues of concern, (2) evaluating movement, (3) discussing treatment adjustments, (4) getting an understanding of what to expect, and (5) getting inappropriate medication revised. |
| 1. Wuthrich, et al. 2. 2019 3. RCT 4. Australia | To determine the feasibility, acceptability and initial efficacy of telephone-delivered cognitive behavioral therapy (CBT) for the treatment of anxiety and depressive symptoms in people with Parkinson’s disease. | 1. Patients and caregivers (n=11) 2. Setting: home | Clinical psychologist provided consultation. | The program consisted of 10 weekly manualized sessions (workbook) supported by 45-minute telephone consultations with an intern clinical psychologist using a therapist manual. The program included core CBT skills to reduce anxiety and depression including psychoeducation, training in activity scheduling, mood monitoring, thought challenging, and communication skills. |
| 1. Fleisher, et al. 2. 2020 3. Case study 4. USA | This study describe the first two iterations of this model and provide two case studies to illustrate the challenges and opportunities of this approach. | 1. Patients(n=52, age: 78.6±7.7) 2. Caregivers (n=52, age: 66.5 ±11.3) 3. Setting: home | A multidisciplinary team included social worker, registered nurse, movement disorder specialist, research collaborator. | Social worker: psychosocial needs assessment of dyad, referrals to community resources. registered nurse: vitals, medication reconciliation, home safety assessment, education & counseling, interim phone follow up. Movement disorder specialist: telehealth visit, referral, pharmacologic management, education and counseling. Research collaborator: data collection, Technology & telehealth set up. |
| 1. Jordan, et al. 2. 2020 3. Qualitative study 4. USA, Canada | This is a secondary analysis of a large, multi-site randomized clinical trial of interdisciplinary outpatient neuropalliative care. We aims to explores patient and care partner needs related to future planning. | 1. Patients (n=30, age: 66±8) 2. Caregivers (n=30, age: 68±7) 3. Setting: outpatient | An outpatient interdisciplinary palliative care team consisted of a neurologist with palliative care experience, a nurse, a social worker, and a chaplain. | Interview topics included future planning, planning in the context of potential cognitive changes or dementia, communication about the future with spouses/care partners/family members, and perceptions of illness progression. |
| 1. Kluger, et al. 2. 2020 3. RCT 4. USA | To determine if outpatient PC is associated with improvements in patient-centered outcomes compared with standard care among patients with PDRD and their caregivers. | 1. Patients(n=210, age: 70.1±8.2) 2. Caregivers (n=175, age: 66.1±11.1) 3. Setting: outpatient | Outpatient integrated PC administered by a neurologist, social worker, chaplain, and nurse using PC checklists, with guidance and selective involvement from a palliative medicine specialist. | Palliative medicine specialists primarily focused on the complex goals of care discussions and symptom management. The typical visit duration was 2 to 2.5 hours and addressed nonmotor symptoms, goals of care, anticipatory guidance, difficult emotions, and caregiver support. |
| 1. Tamplin, et al. 2. 2020 3. RCT 4. Australia | To analyse the effects of ParkinSong group singing sessions on Parkinson’s communication and wellbeing outcomes for people with PD and caregivers over 12 months. | 1. Patients (n=75) 2. Caregivers (n=44) 3. Setting: community | All group facilitators received training in the  ParkinSong protocol with regular fidelity checking  from the research team. Weekly sessions were facilitated by a music therapist, speech pathologist and  allied health assistant. | The 2-hour ParkinSong session protocol included 30 minutes of vocal warm-ups, exercises and activities designed to develop and extend respiratory strength and control, vocal loudness, articulation, pitch control, and communication confidence, and address sensory processing deficits. The communication strategies were taught and practised to increase vocal loudness and to improve self-monitoring of communication and internal cueing. |
| 1. Gao, et al. 2. 2021 3. Pilot study 4. China | The specific goal of the pilot study is to test the overall effect of medication management and rehabilitation training based on the Care-PD platform in a home environment | 1. Patients and caregivers (n=56) 2. Setting: home | An expert team composed of occupational therapists, graphic designers, and information technology experts has developed a mobile application called Care-PD, which has been tested and explored in reality. | The Care-PD program is a service platform for PD medication management and rehabilitation training. It is committed to providing individualized medication management, symptom monitoring, professional counseling, and home life and community communication services for persons with PD. |
| 1. Lyons, et al. 2. 2021 3. Quasi-experimental study 4. Australia | To explore health benefits for couples participating together in an existing community-based self-management workshop for Parkinson’s disease. | 1. Patients (n=39) 2. Caregivers (n=39) 3. Setting: community | Program leaders were all peer trainees trained by co-principal investigator. The program coordinator, M.G., was also a master trainer who observed at least two sessions for each cohort of workshops and worked closely with all program leaders. | The seventh week was added to the 6-week CDSMP to add PD-specific content to the curriculum. The CDSMP covers various aspects of chronic conditions (e.g., depression, sleep problems, exercise). It also teaches self-management skills of monitoring, taking action, problem-solving, decision-making, and evaluating results. These skills have been shown to develop competence to take charge of one’s health. |
| 1. Prieto, et al. 2. 2021 3. Qualitative study 4. USA | To examine the experiences of people with Parkinson’s and their care partners (CPs) who participated in a Parkinson’s-focused community dance class in a northeastern state of the United States. | 1. Patients (n=5, age: 64±6.9) 2. Caregivers (n=5, age: 70±7.2) 3. Setting: community | Two of the three dance instructors had completed a Dance for PD training workshop from Mark Morris Dance Group. The other dance instructor had extensive professional experience as a performer and dance educator. | The lead author observed the dance classes prior to conducting the interviews to provide further context. Classes lasted between 60 and 120 min and occurred one to four times a week. Classes were held at local dance studios or art spaces. Dance classes were free for all participants, and CPs were encouraged to attend. |
| 1. Fothergill-Misbah, et al. 2. 2021 3. Qualitative study 4. Africa | To explore the role of support groups in the management of PD in Kenya, sub-Saharan Africa. | 1. Patients 2. Caregivers 3. Setting: community | The first author established a new PD support group in Mombasa during the fieldwork period to help address an important information gap identified among PwP and their caregivers. | Monthly meetings were held in the waiting room of a private neurology clinic, at a time that suited the participants; they were also open to those not accessing the clinic. The founder of the PD NGO ran the first meeting where attendees had the opportunity to introduce themselves, meet other members, and discuss future topics. |
| 1. Giguere-Rancourt, et al. 2. 2022 3. RCT 4. Canada | The aim of this study was to test ease of implementation, differential safety and preliminary efficacy of two top-down (strategy-learning) home-based, individualized, cognitive interventions: Goal Management Training (GMT), adapted for PD-MCI (Adapted-GMT), and a psychoeducation program combined with mindfulness exercises (PSYCH-Mind). | 1. Patients (n=12, age: 70.5±4.6) 2. Caregivers (n=11, age: 64.9±6.9) 3. Setting: home | All assessments and interventions were performed by a Senior Ph.D. Candidate under the supervision of a registered neuropsychologist. All sessions were planned beforehand with each participant. | Five modules were designed as a discussion with patients and caregivers about various PD symptoms: module I- brain and motor symptoms; module II- autonomic symptoms; module III- psychological symptoms; module IV- brain and cognition. The mindfulness exercises were practiced in both groups. |
| 1. Schindler, et al. 2. 2022 3. RCT 4. USA | TeleDREAMS, a distance learning version of the Developing a Research Participation Enhancement and Advocacy Training Program for Diverse Seniors (DREAMS) program, provides remote clinical research process and advocacy education to older adults with PD and their care partners. | 1. Patients (n=28, age: 68.1±8.3) 2. Caregivers (n=15, age: 66.7±6.4) 3. Setting: home | A team of faculty, graduate, and undergraduate research assistants designed the TeleDREAMS binder, eight clinical research process and advocacy modules, which was reviewed by multiple investigators and stakeholder and community advisors. | The TeleDREAMS included Week 1: Introduction to Research Advocacy; Week 2: Parkinson Disease Clinical Research in the Pipeline; Week 3: Ethics and Research; Week 4: Analysis and Evaluation of Clinical Research; Week 5: Aging and Clinical Research; Week 6: Understanding Informed Consent and Health Literacy; Week 7: Effective Advocacy in the Clinical Research Process; Week 8: Engaging Diverse Communities. |
| 1. Seritan, et al. 2. 2022 3. Pre-post study 4. USA | To evaluate the feasibility of an online MBCT intervention for people with PD and their caregivers. The second aim was to assess pre- to post-differences in anxiety and depression scores, respectively. | 1. Patients (n=24, age: 62.5 ±9.1) 2. Caregivers (n=4, age: 61.8 ±8.5) 3. Setting: home | Those who chose to participate and signed consent were scheduled for a 90-min online screening interview with the principal investigator, an experienced geriatric psychiatrist. | Group sessions were conducted in Zoom. Home practice was recorded. The final week exercise, in which participants pass a ball of yarn from one to the next and thank each other for their contributions to the group, was conducted without props. |
| 1. Torriani-Pasin, et al. 2. 2022 3. RCT 4. Brazil | The primary aim of this study was to evaluate the adherence rate, barriers to attend and safety of a telemonitoring program for individuals with PD; the secondary aim was to assess the individuals, and their family member's, perceived overall experience of performing such a telemonitoring‐based physical exercise program. | 1. Patients (n=19, age: 69.80 ± 10.12) 2. Caregivers (n=6, age: 62.35 ± 22.31) 3. Setting: home | The team leader and the physical education instructor should weekly meetings with the team members. The health instructors' team was in charge of monitoring the interns. The interns' team was in charge of recording the videos and submit them for approval by the team leader and physical. | The elemonitoring‐based physical exercise program included two exercise videos. The contents included warm up, balance, aerobic capacity, resistance training, transfers, and cool down. |
| 1. Fleisher, et al. 2. 2023 3. Non-RCT 4. USA | To evaluate the feasibility and efficacy of four structured, interdisciplinary, telehealth-enhanced home visits for homebound PWPD-caregiver dyads over approximately one year. | 1. Patients (n=384, age: 69.1±8.8) 2. Caregivers (n=94, age: 70.1±7.8) 3. Setting: home | IN-HOME-PD dyads received four protocol-driven home visits from a nurse and a social worker, accompanied by telehealth connection with a movement disorders specialist, over one year. | Peer mentors were asked to speak with their mentees for 30 min weekly by phone, videoconference, or in person. Mentors could focus on topics of mentee concern and refer to the Share the Care handbook to guide discussions. Participants maintained brief study diaries documenting the date, duration, utility of each conversation, and any concerns. Mentors were invited to quarterly conference calls to share their experiences with each other and the study team. |

### 1.3 Supplementary Table S3. PRISMA-ScR

| **SECTION** | **ITEM** | **PRISMA-ScR CHECKLIST ITEM** | **REPORTED ON PAGE #** |
| --- | --- | --- | --- |
| **TITLE** | | | |
| Title | 1 | Identify the report as a scoping review. | Page 1 |
| **ABSTRACT** | | | |
| Structured summary | 2 | Provide a structured summary that includes (as applicable): background, objectives, eligibility criteria, sources of evidence, charting methods, results, and conclusions that relate to the review questions and objectives. | Page 1 |
| **INTRODUCTION** | | | |
| Rationale | 3 | Describe the rationale for the review in the context of what is already known. Explain why the review questions/objectives lend themselves to a scoping review approach. | Page 2 |
| Objectives | 4 | Provide an explicit statement of the questions and objectives being addressed with reference to their key elements (e.g., population or participants, concepts, and context) or other relevant key elements used to conceptualize the review questions and/or objectives. | Page 2 |
| **METHODS** | | | |
| Protocol and registration | 5 | Indicate whether a review protocol exists; state if and where it can be accessed (e.g., a Web address); and if available, provide registration information, including the registration number. | None |
| Eligibility criteria | 6 | Specify characteristics of the sources of evidence used as eligibility criteria (e.g., years considered, language, and publication status), and provide a rationale. | Page 3 |
| Information sources* | 7 | Describe all information sources in the search (e.g., databases with dates of coverage and contact with authors to identify additional sources), as well as the date the most recent search was executed. | Page 3 |
| Search | 8 | Present the full electronic search strategy for at least 1 database, including any limits used, such that it could be repeated. | Supplementary Table 1 |
| Selection of sources of evidence† | 9 | State the process for selecting sources of evidence (i.e., screening and eligibility) included in the scoping review. | Page 3-4 |
| Data charting process‡ | 10 | Describe the methods of charting data from the included sources of evidence (e.g., calibrated forms or forms that have been tested by the team before their use, and whether data charting was done independently or in duplicate) and any processes for obtaining and confirming data from investigators. | Page 3-4 |
| Data items | 11 | List and define all variables for which data were sought and any assumptions and simplifications made. | Page 3-4 |
| Critical appraisal of individual sources of evidence§ | 12 | If done, provide a rationale for conducting a critical appraisal of included sources of evidence; describe the methods used and how this information was used in any data synthesis (if appropriate). | None |
| Synthesis of results | 13 | Describe the methods of handling and summarizing the data that were charted. | Page 3-4 |
| **RESULTS** | | | |
| Selection of sources of evidence | 14 | Give numbers of sources of evidence screened, assessed for eligibility, and included in the review, with reasons for exclusions at each stage, ideally using a flow diagram. | Figure 1 |
| Characteristics of sources of evidence | 15 | For each source of evidence, present characteristics for which data were charted and provide the citations. | Page 4  Supplementary Table 2 |
| Critical appraisal within sources of evidence | 16 | If done, present data on critical appraisal of included sources of evidence (see item 12). | None |
| Results of individual sources of evidence | 17 | For each included source of evidence, present the relevant data that were charted that relate to the review questions and objectives. | Page 4-6 |
| Synthesis of results | 18 | Summarize and/or present the charting results as they relate to the review questions and objectives. | Page 4-6 |
| **DISCUSSION** | | | |
| Summary of evidence | 19 | Summarize the main results (including an overview of concepts, themes, and types of evidence available), link to the review questions and objectives, and consider the relevance to key groups. | Page 6-7 |
| Limitations | 20 | Discuss the limitations of the scoping review process. | Page 8 |
| Conclusions | 21 | Provide a general interpretation of the results with respect to the review questions and objectives, as well as potential implications and/or next steps. | Page 8 |
| **FUNDING** | | | |
| Funding | 22 | Describe sources of funding for the included sources of evidence, as well as sources of funding for the scoping review. Describe the role of the funders of the scoping review. | Title Page |
